# IARNN-Based Semantic-Containing Double-Level Embedding Bi-LSTM for Question-and-Answer Matching

**DOI:** 10.1155/2019/6074840

**Published:** 2019-03-03

**Authors:** Chang-zhu Xiong, Minglian Su

**Affiliations:** College of Electronics and Information Engineering, Sichuan University, Chengdu 610065, China

## Abstract

We propose a novel end-to-end approach, namely, the semantic-containing double-level embedding Bi-LSTM model (SCDE-Bi-LSTM), to solve the three key problems of Q&A matching in the Chinese medical field. In the similarity calculation of the Q&A core module, we propose a text similarity calculation method that contains semantic information, to solve the problem that previous Q&A methods do not incorporate the deep information of a sentence into the similarity calculations. For the sentence vector representation module, we present a double-level embedding sentence representation method to reduce the error caused by Chinese medical word segmentation. In addition, due to the problem of the attention mechanism tending to cause backward deviation of the features, we propose an improved algorithm based on Bi-LSTM in the feature extraction stage. The Q&A framework proposed in this paper not only retains important timing features but also loses low-frequency features and noise. Additionally, it is applicable to different domains. To verify the framework, extensive Chinese medical Q&A corpora are created. We run several state-of-the-art Q&A methods as contrastive experiments on the medical corpora and the current popular insuranceQA dataset under different performance measures. The experimental results on the medical corpora show that our framework significantly outperforms several strong baselines and achieves an improvement of top-1 accuracy of up to 14%, reaching 79.15%.

## 1. Introduction

Question answer selection is a subdirection in the field of question answering systems. With the development of natural language processing, question answering systems have been at the forefront of artificial intelligence research. For example, Apple's Siri and Microsoft's Cortana can provide humans with a good operating experience in human-computer interaction. In general, the question answering system is divided into task robots, chat robots, and solution robots according to different application fields. In this paper, the Chinese medical question-and-answer matching is performed by a solution robot.

Due to limited medical resources, increasing number of people tend to obtain medical information online. For this reason, Q&A selection in the medical field has a promising future. For example, YouWenBiDa (http://www.120ask.com) provides an extensive medical Q&A information database for patients. Based on these massive resources, many meaningful studies related to medical Q&A are being carried out. Sadikin et al. [1] utilized data features to extract drug name entities from medical texts; Goodwin and Harabagiu [2] created clinical decision support based on a medical Q&A system; Zhang et al. [3] proposed end-to-end character-level multiscale CNNs for medical Q&A selection. Based on previous research, we propose an SCDE-Bi-LSTM algorithm that greatly improves the accuracy of Q&A matching in the Chinese medical field.

In this study, we performed many experiments to verify our novel model. A total of 60,000 question samples and 112,986 answer samples were collected from http://www.120ask.com, with 2 correct answers to each medical question. The data resources used throughout this paper are available on GitHub (https://github.com/Vitas-Xiong/Chinese-Medical-Question-Answering-System), including the text of the original Q&A statements. The corpora that we created contain most of the contents of the medical questions and answers, so we can make full use of the Q&A corpora to choose the best answers for medical questions.

In the process of Q&A pair matching, for the given question (q) and answer pool {*a*_1_, *a*_2_, …, *a*_*l*_} (*l* is the number of answers in the answer pool, including at least one correct answer), the correct answer (*a*_*n*_) (1 ≤ *n* ≤ 1) related to the question (q) in the answer pool needs to be retrieved.  Table 1 is a sample of a Q&A pair in the database created in this paper.

**Figure 1:**
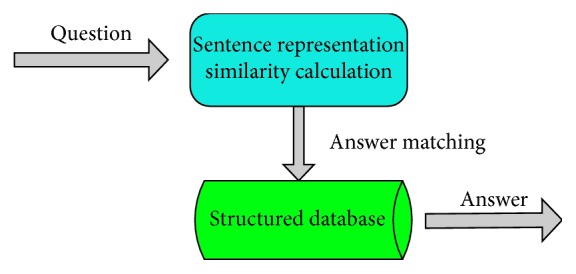
The simplified process of answer selection.

As shown in Table 1, a Q&A pair contains one answer and one question (q is the question, a+ is the correct answer, and a− is the wrong answer). The purpose of the model is to maximize the similarity between the question and the correct answer and minimize the similarity between the question and the wrong answer.  Figure 1 shows the simplified process of answer selection.

As shown in Figure 1, the simplification process of answer selection consists of the following five steps: (1) question input; (2) sentence representation vector initialization; (3) Q&A statement similarity calculation; (4) answer statement similarity ordering in the structured database; and (5) output of the answer with the highest similarity.

To more accurately match the correct answer (a+) of q from the answer pool, the improved SCDE-Bi-LSTM is proposed to select the best answer in the Chinese medical Q&A corpora. The model inputs are representative vectors of the questions and the positive and negative answers. The features of each sentence are calculated through the neural network, and the model then outputs the similarity difference between the question and the answer. The objective function is to ensure that the similarity interval is the largest. When a user inputs a question, the system will output the most suitable answer.

In short, this paper mainly contributes to the following aspects:

- We are the first to achieve Chinese medical Q&A matching close to 80% accuracy, up to 14% above the baseline.
- We create and release an extensive Chinese medical Q&A database through web resources. This database is publicly available.
- We present an improved Bi-LSTM-based algorithm (SCDE-Bi-LSTM) that significantly outperforms several strong baselines under different performance measures.
- In the initialization process of sentence vector representation, we propose a double-level embedding sentence vector expression method to avoid the influence of Chinese word segmentation error on the experimental results.
- We propose a similarity calculation method that contains semantic information, which can calculate similarity at the semantic level.

The above five contributions are novel; the third is an improvement and optimization of the method based on the prior technologies, and the other four are initial innovations.

## 2. Related Work

Traditional Q&A selection systems usually require feature engineering, linguistic tools, or other external resources. Yih et al. [4] extracted semantic features using enhanced lexical semantic models, namely, WordNet; Wang and Manning [5] converted the answer selection into a syntactic matching problem using tree-edit models with structured latent variables; Heilman and Smith [6] presented an improved tree-edit model to recognize answers to questions, and Severyn and Moschitti [7] proposed a model that automatically extracts features for answer selection using parsed trees. Although the above methods have a certain level of effectiveness, they require extra resources and artificial extraction features and use linguistic tools, which increase the complexity of the model.

At present, Q&A research mainly focuses on extracting sentence features automatically using deep learning technology. Feng et al. [8] presented a model that automatically extracted features using a CNN deep learning algorithm with 65.3% accuracy on the insuranceQA dataset (https://github.com/shuzi/insuranceQA). Wang and Nyberg [9] transformed the answer selection task into a classification or sorting problem using the LSTM framework, and Xiong et al. [10] increased the *F*1 value on the Stanford Q&A dataset to 80.4% by using the DCN dynamic collaborative attention network. In addition, Tan et al. [11] proposed an attention-based RNN model to introduce question attention into the answer representation, established the matching of Q&A pairs based on the Bi-LSTM model, and calculated their proximity using cosine similarity. They all implemented word segmentation as the vector initialization part, but the impacts of word segmentation errors were not discussed in their papers. Dong et al. [12] proposed an enhanced multicolumn convolutional neural network to learn the distributed representation of questions and answers from the three aspects of the response path, context, and answer type. Additionally, Li et al. [13] proposed calculating the similarity between two sentences by calculating the similarity between words, and Robertson and Zaragoza [14] utilized BM25 values to retrieve multiple answers from a prebuilt corpus. Wang et al. [15] added the inner attention before the hidden layer of the RNN, which performed well in sentence representation and answer selection, and Santos et al. [16] proposed an attentive pooling bidirectional attention mechanism with feature weighting for both questions and answers. Although the above methods improve the accuracy of Q&A matching through feature engineering, attention mechanisms, etc., the problems of semantic level information and Chinese word segmentation error are still not solved.

In addition, there are many techniques in common with image question answering. Guo et al. [17] proposed a TS-LSTM method, which can systematically exploit spatial and temporal dynamics within video sequences; Song et al. [18] proposed a generative approach, referred to as a multimodal stochastic RNNs (MS-RNNs); and Gao et al. [19] proposed a novel end-to-end framework named aLSTMs, an attention-based LSTM model with semantic consistency, to transfer videos to natural sentences. In the field of visual Q&A, there are some explorations of multimodal and multiview approaches. Liu et al. [20] systematically presented a method for MCG generation that is composed of cliques, which consist of neighbor nodes in multimodal feature space and hyperedges that link pairwise cliques; Gao et al. [21] presented a method that can jointly learn the visual features from multiple views of a 3D model and optimize towards the object retrieval task; and Gao et al. [22] designed a multiview discriminative and structured dictionary learning with group sparsity and graph model (GM-GS-DSDL) to fuse different views and recognize human actions. The methods of image Q&A can be used as a reference for text Q&A, especially in neural networks, and there are certain innovations in these papers. However, the key problem of similarity calculation in the Q&A matching has not been solved.

**Figure 2:**
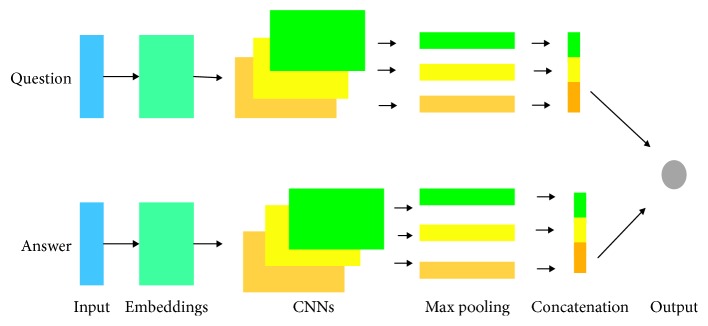
The framework of convolutional neural networks.

Among the different applications of the above Q&A systems, the CNN framework is currently popular due to its ability to focus on extracting local information of features. Qiu and Huang [23] constructed a community-based question answering system using convolutional neural networks. Yin et al. [24] proposed a machine comprehension model through a convolution neural network based on an attention mechanism. The advantage of the CNN framework is that it can realize convolution and capture the local information of the text.  Figure 2 shows the simplified process of the CNN extraction feature.

As shown in Figure 2, the question and answer features are extracted separately by the CNN network and then concatenated. However, for Q&A statements containing sequence information, CNN has no advantage in extracting the internal correlation features of sentences. LSTM can be widely used in the field of text Q&A because it can take the sequential relationship between words into account. Yang et al. [25] constructed an online time-series prediction system using LSTM, which has strong adaptability and robustness.

## 3. Methodology

**Figure 3:**
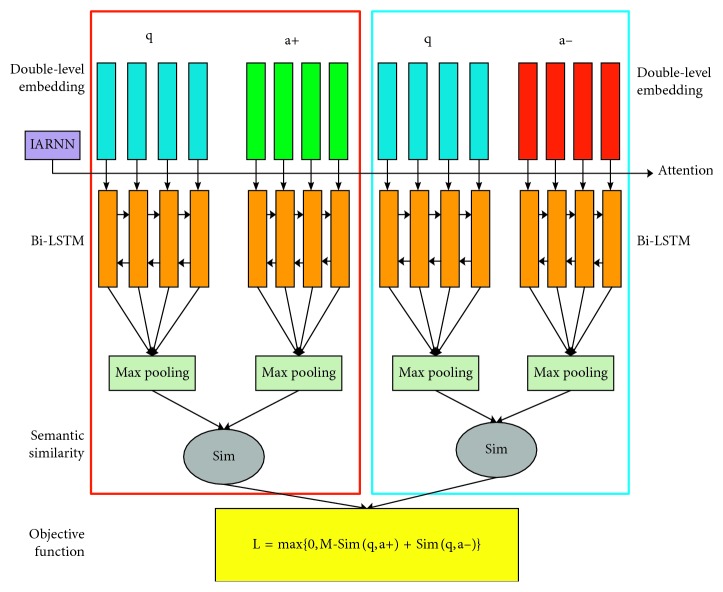
Question answering matching model framework.

In this section, we introduce our novel semantic-containing double-level embedding Bi-LSTM model (SCDE-Bi-LSTM) based on the inner attention-based RNN (IARNN) attention mechanism.  Figure 3 is the overall framework of the question-and-answer matching model that we proposed.

As shown in Figure 3, q is a medical question statement, a+ is the correct answer, and a− is the wrong answer. We propose an improved fixed-length LSTM that shares the weight of all parameters, so we need to truncate or supplement the Q&A statement first to make the sentences consistent in length. In addition, we adopt the improved IARNN attention mechanism before the extraction of sentence features to avoid backward deviation of the features. After that, the time sequence information processed by the attention mechanism is input into the Bi-LSTM model. After max pooling, the sequence features are selected by LSTM. When training a neural network, similarity is calculated in (q, a+) and (q, a−); then, the cost function is calculated. The goal is to make the positive sample similarity as high as possible and the negative sample similarity as low as possible. When testing the neural network, we need to calculate the similarity between q and each candidate answer and record the most similar candidate answer as the best answer. If the best answer happens to be in ground truth, the question is successfully retrieved and counted among the top-1 accuracy.

### 3.1. Double-Level Embedding Vector Representation

**Figure 4:**
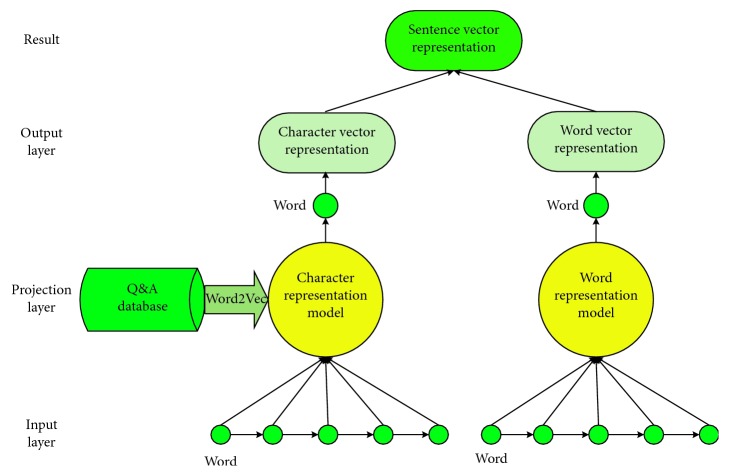
The process of sentence vector representation.

The Q&A sentence vector representation is an important step in the generation of text features. Utilizing the LSTM network to process matching tasks requires obtaining a vector representation of the sentence. Due to unrecorded words in the medical field, word segmentation errors will have a great impact on the results of this experiment. Gensim is implemented for character-level embedding and word-level embedding after word segmentation.  Figure 4 shows the double-level embedding vector representation method based on two improved models, which can successfully reduce the experimental error caused by Chinese medical word segmentation.

As shown in Figure 4, after word segmentation on all Q&A sentences, we perform word and character vector training to obtain a training model for all words and characters by Word2Vec. After duplicate removal, 5004 Chinese characters and 38745 Chinese words are obtained. By using the word embedding and character embedding vector representation model, a representative dictionary of these character vectors and word vectors is obtained. Finally, based on each word and character in a sentence, we can obtain two kinds of vector representations of each sentence by strategically combining each word and character in the sentence. In this experiment, each word is represented by 100-dimensional vectors and the window size is set to 5. The model is introduced by the embedded vector, and the eigenvector representation of the word is obtained after splicing.

Because the length of the word embedding and the character embedding is inconsistent, we first adopt the zero vector to complement the word embedding. By weighting two levels of vector representation, the final sentence vector of the improved model is represented by Sen:

(1) $$\begin{array}{c} \text{Sen}=\alpha \;*\;{\text{Sen}}_{\text{word}}+\beta \;*\;{\text{Sen}}_{\text{character}}, \end{array}$$

where Sen_word_ represents the word embedding vector representation and Sen_character_ represents the character embedding vector representation. The sum of *α* and *β* is a constant of 1, and we set *α* as 0.6 in this paper. Sentences are represented by 100-dimensional vectors through double-level embeddings, and features of the sentence vectors are then extracted by the enhanced attention mechanism.

### 3.2. IARNN Attention Mechanism

**Figure 5:**
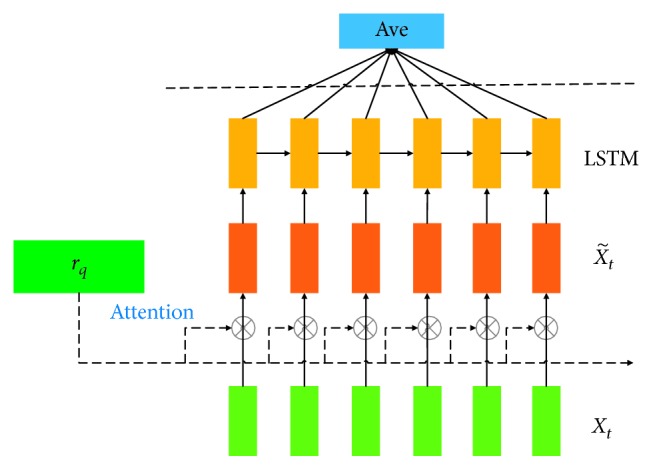
The structure of the IARNN attention mechanism.

There may be synergies between words in the fixed sentences, which reduces the accuracy of the model in the test set. In addition, since the RNNs focus on timing features, the neural network model at time *t* contains sequence information from all previous moments. When an attention mechanism is added to the RNN framework to obtain more weighted information, the text features near the end of sentences can be selected more easily because the framework contains more previous information, which can lead to backward deviation and weight bias of features. To solve the above problems, the improved IARNN attention mechanism is implemented before the feature extraction process.  Figure 5 shows the process of the IARNN attention mechanism in calculating the timing information of sentences.

As shown in Figure 5, before LSTM training, the IARNN attention mechanism extracts the timing information of *x*_*t*_, which represents a sentence. The algorithm calculates the average feature output at each time as the last output to avoid the loss of feature information. Max pooling is implemented in this process, which adds the weight of the IARNN attention mechanism at each moment. After the IARNN mechanism *α*_t_ is calculated, we obtain output ${\tilde{x}}_{t}$ as follows:

(2) $$\begin{array}{c} {\tilde{x}}_{t}=\alpha_{t}\;*\;x_{t}, \end{array}$$

where *x*_*t*_ is the original input timing sequence feature vector at time *t*, and *α*_*t*_ is defined as follows:

(3) $$\begin{array}{c} \alpha_{t}=\sigma \left(r_{q}^{T}M_{\text{qi}}x_{t}\right), \end{array}$$

where *σ* is a sigmoid function, so the value of *α*_*t*_ is fixed between 0 and 1; *r*_*q*_ is the weight of hidden layers on the attention mechanism; and *M*_qi_ is an attention matrix that converts a Q&A vector representation into a word embedding space.

### 3.3. Multilayer Bi-LSTM Neural Network Model

An RNN (recurrent neural network) is a time-series network structure that can store historical states. However, due to gradient disappearance and gradient descent, multilayer RNN is limited when calculating context information. LSTM is a variation of the RNN that mainly mitigates the problem of RNN long-distance gradient calculations. In the LSTM structure, an input gate I, an output gate O, and a forget gate F are included; additionally, a memory unit C is provided to store information. Gate I can make the input change the state of C; gate O can make C affect the output; and gate F can make C save or discard the previous state information. The input sequence at time *t* is defined as *X*(*t*), which is a 100-dimensional word representation vector. When the hidden layer vector is *h*(*t*), the state update at time *t* is as follows:

(4) $$\begin{array}{c} i_{t}=\sigma \left(W_{i}x\left(t\right)+U_{i}h\left(t-1\right)+b_{i}\right),\\ f_{t}=\sigma \left(W_{f}x\left(t\right)+U_{f}h\left(t-1\right)+b_{f}\right),\\ o_{t}=\sigma \left(W_{o}x\left(t\right)+U_{o}h\left(t-1\right)+b_{o}\right),\\ {\tilde{C}}_{t}=\tanh\left(W_{c}x\left(t\right)+U_{c}h\left(t-1\right)+b_{c}\right),\\ C_{t}=i_{t}\;*\;{\tilde{C}}_{t}+f_{t}\;*\;C_{t-1},\\ h_{t}=o_{t}\;*\;\tanh\left(C_{t}\right), \end{array}$$

where *i*_*t*_/*f*_*t*_/*o*_*t*_/*C*_*t*_ is the input gate value, the forget gate value, the output gate value, and the memory cell value, respectively; *σ* is the sigmoid function; and *W*, *U*, and *R* are the parameters of the LSTM neural network.

**Figure 6:**
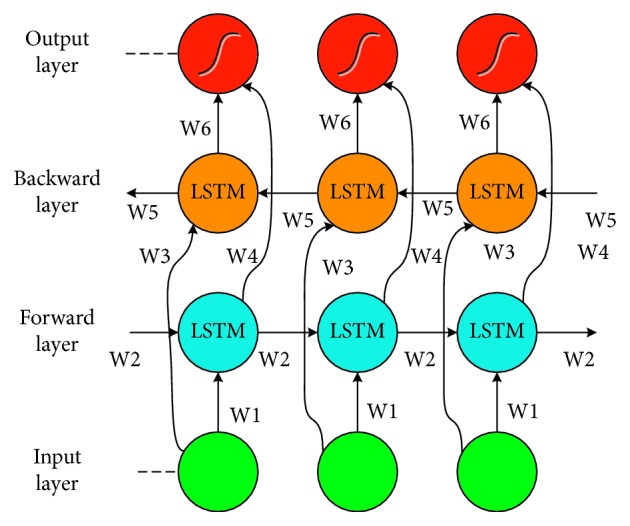
The structure of Bi-LSTM.

The improved Bi-LSTM-based IARNN can solve the problem of the inability of unidirectional LSTM to calculate the context information of the reverse sequence. The forward sequence and the reverse sequence are combined to obtain output *r*_*t*_:

(5) $$\begin{array}{c} r_{t}={\vec{h}}_{t}||{\overset{↼}{h}}_{t}, \end{array}$$

where $\vec{h}$ and $\overset{↼}{h}$ are the calculated results of the Bi-LSTM forward and reverse hidden layers, respectively.  Figure 6 shows a schematic diagram of Bi-LSTM, which is calculated and updated in two directions.

The two training sequences of this bidirectional LSTM structure are directly connected to the output layer, providing complete past and future context status information for each word.

### 3.4. Objective Function and Similarity Calculation

**Figure 7:**
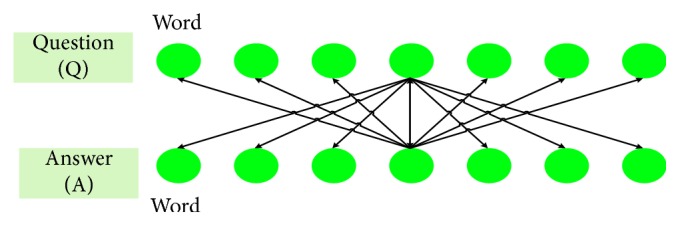
The process of semantic similarity calculation.

The trained neural network model can maximize the similarity between the question and the correct answer and minimize the similarity between the question and the wrong answer. The goal is to maximize the difference between the positive and negative samples. The other Q&A systems generally calculate only the cosine similarity between the vectors and do not involve the depth similarity calculation at the semantic level, which has considerable limitations. Therefore, we propose a similarity calculation containing semantics to define an objective function:

(6) $$\begin{array}{c} L=\max\left\{0,\;\;M-\text{Sim}\left(q,\;\;a+\right)+\text{Sim}\left(q,\;\;a-\right)\right\}, \end{array}$$

where *M* is the maximum interval value, which is set as 0.1, and Sim is the joint similarity calculation method of the Q&A statement and is defined as follows:

(7) $$\begin{array}{c} \text{Sim}\left(q,\;\;a\right)=\theta_{1}\;*\;{\text{Sim}}_{\text{semantic}}\left(q,\;\;a\right)+\theta_{2}\;*\;{\text{Sim}}_{\text{text}}\left(q,\;\;a\right), \end{array}$$

where Sim_text_ is the cosine similarity calculation method of the vector and Sim_semantic_ is the semantic similarity calculation method of the vector. The sum of *θ*_1_ and *θ*_2_ is a constant of 1, and we set *θ*_1_ as 0.6 in this paper.  Figure 7 shows the simplified process of the semantic similarity calculation method.

As shown in Figure 7, the two rows of spheres represent question and answer statements respectively and each sphere represents a word. The semantic similarity calculation method is explained as follows: there are *m* word vectors in question statement *Q*, which are *q*_1_, *q*_2_, …, *q*_*m*_; there are *n* word vectors in answer statement A, which are *a*_1_, *a*_2_, …, *a*_*n*_. First, the cosine similarities between *q*_1_ and *a*_1_ to *a*_*n*_ are calculated, and the word similarity value with the largest similarity between *q*_1_ and A is recorded; then, we calculate the maximum similarity value of *q*_2_ and the sum of the maximum similarity values. For sentence A, we also record the maximum similarity value of *a*_1_, *a*_2_, …, *a*_*n*_ in *Q* and calculate the sum of the maximum similarity value in A. Finally, the sum of the two maximum similarities is divided by the sum of the lengths of two sentences to obtain the semantic similarity between *Q* and A. There are explanations as follows:

(8) $$\begin{array}{c} {\text{Sim}}_{\text{semantic}}=\frac{\sum_{i=1}^{m}Q_{\max}+\sum_{i=1}^{n}A_{\max}}{m+n}, \end{array}$$

where *Q*_max_ is the sum of the maximum similarity of each word in the question sentence and *A*_max_ is the sum of the maximum similarity of each word in the answer sentence:

(9) $$\begin{array}{c} Q_{\max}=\text{Max}\left(\text{cosine}\left(q_{i},\;\;a_{1}\right),\;\ldots ,\text{ cosine}\left(q_{i},\;\;a_{n}\right)\right),\\ A_{\max}=\text{Max}\left(\text{cosine}\left(q_{1},\;\;a_{i}\right),\;\ldots ,\text{ cosine}\left(q_{m},\;\;a_{i}\right)\right),\\ {\text{Sim}}_{\text{text}}\left(q,\;\;a\right)=\text{cosine}\left(q,\;\;a\right)=\frac{\left\|q\cdot a\right\|}{\left\|q\right\|\cdot \left\|a\right\|}. \end{array}$$

To avoid the problem of local optimal solution, we select Adam as the optimizer. In the Bi-LSTM layer, we perform dropout operation to avoid the problem of overfitting.

## 4. Experiments and Results

### 4.1. Datasets

The data used in this article are collected from the Internet. The data resources are all on GitHub (https://github.com/Vitas-Xiong/Chinese-Medical-Question-Answering-System). The database contains 60,000 question samples and 112,986 answer samples and contains the largest quantity of originally collected data. The average length of the question statement is 50 characters, and the average length of the answer statement is 70 characters. Each question has an average of 2 correct answers in the entire answer pool. In the train set, we choose 50,000 questions to form 300,000 train samples, of which 50,000 are positive samples and 250,000 are negative samples, with one positive sample and five negative samples for each question. A positive sample is a pairing of a question and its correct answer. A negative sample is a pairing of a question and a wrong answer that is randomly selected from 104,583 answers. In the development set and the test set, the remaining 10,000 questions are utilized to build 1,000,000 samples, of which 10,000 are positive samples and 990,000 are negative samples, with one positive sample and ninety-nine negative samples for each question. Additionally, the development set and the test set are of the same size. We set the size of the answer pool for each question to be 100, and the top-1 accuracies are recorded based on each answer pool. We adopt the top-*k* accuracy and loss on the test/train set as the evaluation criteria of the models.

### 4.2. Baselines

We conducted eight comparative experiments, as shown below.

#### 4.2.1. BM25

Robertson and Zaragoza [14] utilized BM25 as a search relevance score. The main idea is to perform morpheme parsing on query and generate the morpheme qi. Then, for each search result D, the correlation score between each morpheme qi and D is calculated. Finally, the correlation score between query and D is obtained by the sum of the correlation scores of qi with respect to D.

Word embeddings: in the pretraining word vector phase, we conducted comparative experiments using word embeddings. We performed Chinese word segmentation and word vector training using Gensim.

#### 4.2.2. Character Embeddings

The vector representation method of character embeddings does not consider the sequence information of the sentence during the initialization of the sentence.

#### 4.2.3. Cosine Similarity Method

The cosine similarity method is used as a controlled experiment in the similarity calculation module.

#### 4.2.4. Attentive-Pooling Networks

We implemented the attentive pooling mechanism proposed by Santos et al. [16] as contrastive experiments. It can apply attention mechanisms to question and answer statements at the same time.

#### 4.2.5. DMN (Dynamic Memory Network)

Kumar et al. [26] constructed an improved question answering system through DMN, which is mainly utilized to process input sequences and can perform end-to-end training.

#### 4.2.6. Multiscale CNN

Zhang et al. [3] proposed end-to-end character-level multiscale CNNs for medical Q&A selection. We set the multiscale CNN as a baseline for the control experiment in this paper.

#### 4.2.7. CapsNet

Wang et al. [27] proposed an attention-based Bi-GRU-CapsNet model for hypernymy detection between compound entities that adopted a new “vector in vector out” delivery scheme in which the input and output of neurons are vectors.

#### 4.2.8. ESIM + ELMo

Peters et al. [28] proposed a new type of deep contextualized word representation in which the word vectors are learned functions of the internal states of a deep bidirectional language model (biLM).

#### 4.2.9. Multiview

Zhou et al. [29] proposed a multiview response selection model that integrates information from two different views, i.e., word sequence view and utterance sequence view.

### 4.3. Experimental Settings

The models presented in our paper are implemented by the Python programming language and TensorFlow neural network framework. The Jieba and Gensim tools are used for word segmentation and word vector pretraining. The word vector pretraining window is set to 5, and the vector dimension is set to 100. Additionally, we set the length of the question statement to 50 and the length of the answer statement to 70. The maximum interval value *M* of the objective function is set to 0.1.

In terms of neural network hyperparameter settings, we select Adam as the optimizer in the Bi-LSTM network and the number of LSTM layers is set to two. The value of our Dropout hyperparameter is set as 0.5; the number of hidden layer nodes is set as 200; the learning rate is set as 0.1; and the LSTM output features are selected by max pooling. A batch is set as 50, and the model is trained for a total of 150 epoches.

### 4.4. Results

In the pretraining phase of the word vector, we performed a series of comparative experiments on character embedding, word embeddings, and double-level co-occurrence embedding.  Table 2 shows the results of the experiments.

As shown in Table 2, the accuracy of double-level vector on the train set is 1 to 4 percentage points higher than that of the other models. The loss function of double-level embedding performs better, and its accuracy on the test set is 2 to 3 percentage points higher than that of other approaches. Thus, the improved double-level embedding can solve the problem of segmentation error and loss of sequence information. Comparative experiments on different similarity calculations are shown in Table 3.

As shown in Table 3, the semantic similarity calculation module is 3 percentage points higher than the cosine similarity calculation on the train set and 8 percentage points higher on the test set. The experimental results show that the semantic similarity calculation module has advantages over cosine calculations.

Table 4 shows the comparative experimental results of applying the model to the insuranceQA dataset.

As shown in Table 4, the model also has good performance and generalizability in other Q&A datasets.

The experimental results of SCDE-Bi-LSTM and the other 6 comparative models are shown in Table 5.

As shown in Table 5, the model proposed in this paper shows great advantages and improved performance in comparison with other current state-of-the-art methods. To verify the validity of SCDE-Bi-LSTM under different application requirements, we adopt the *F*1 value, recall, and top-2/3 accuracies on the test set as our performance measures.

As shown in Table 6, the SCDE-Bi-LSTM outperforms several strong models, regardless of the *k* value of top-*k* accuracy. The SCDE-Bi-LSTM also performs well under *F*1 and recall rates, showing that the novel end-to-end approach that we proposed is effective and robust under different performance measures.

**Figure 8:**
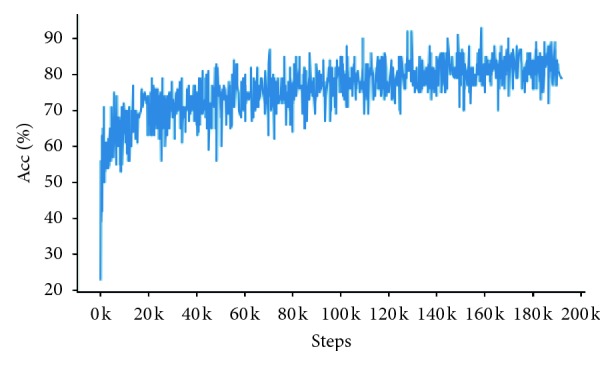
Top-1 accuracy curve of CapsNet on the train set.

**Figure 9:**
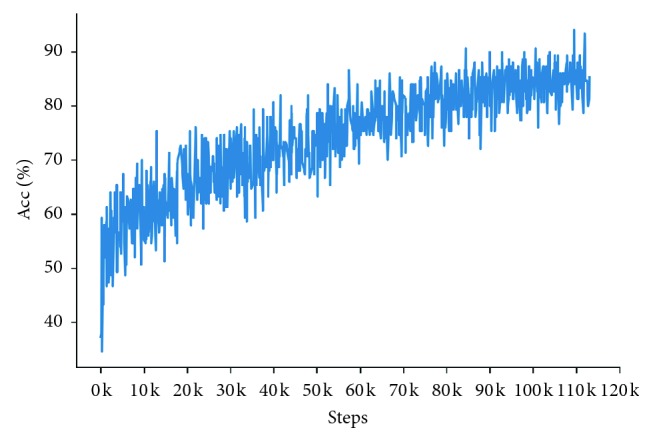
Top-1 accuracy curve of the SCDE-Bi-LSTM model on the train set.

Figures 8 and 9 show the comparison of CapsNet and SCDE-Bi-LSTM based on the top-1 accuracy curve on the train set. They illustrate changes in the top-1 accuracy with the training of models.

As shown in Figures 8 and 9, in comparison with state-of-the-art models, the SCDE-Bi-LSTM model achieves higher accuracy with fewer training times and has an upward trend throughout the training process.

To verify the validity of SCDE-Bi-LSTM, we propose a novel evaluation standard for steps. When a model reaches 50% of the top-1 accuracy for the first time on the train set, the training steps are recorded. A model with fewer steps can extract useful information more quickly from the question and answer statements.  Table 7 shows the number of steps taken by models, when they first achieve 50% accuracy.

As shown in Table 7, SCDE-Bi-LSTM can achieve 50% accuracy with the fewest steps. From this aspect, the method is effective and has higher sensitivity to Q&A statements.

## 5. Conclusions

The framework proposed in this paper greatly improves the accuracy of question-and-answer matching in the Chinese medical field. We concentrate on solving three main problems in Q&A matching. To solve them, this paper presents a Bi-LSTM model with double-level co-occurrence embedding containing semantic information based on the IARNN mechanism. The experiment shows that the novel end-to-end approach that we propose outperforms several strong state-of-the-art Q&A methods under different performance measures. The accuracy of top-1 on the test set can reach 79.15%, and the loss of the train set can be reduced to 0.95, which can improve the accuracy by 14% compared with the multiscale CNN baseline. On other datasets, we also achieved the best performance compared to other models. In addition, we adopted *F*1 value, recall, and three top-k accuracies to verify the validity of SCDE-Bi-LSTM, and the experimental results show that SCDE-Bi-LSTM is robust and effective under different performance measures. Finally, we present a new performance measure to verify that SCDE-Bi-LSTM is more efficient in the extraction of statement information than several other strong state-of-the-art methods. Therefore, the research in this paper may have a significant medical application impact and practical value.

In the future, we will further evaluate the models proposed using different question answering systems, such as paragraph-based answer content prediction. In addition, we will increase the amount of data to further verify the performance of SCDE-Bi-LSTM on different databases.

## Figures and Tables

**Table 1:** The form of a Q&A pair in the database.

(q) 六味地黄丸、前列康胶囊、阿莫西林胶囊, 这三种药可以一起服用吗? (Can Liuwei Dihuang Wan, Qianliekang capsules, and amoxicillin capsules be taken together?)

(a+) 可以的。没有问题的, 最好是遵医嘱在医生的指导下治疗. (Yes, it is ok. It's better to follow a doctor's advice and be treated under the guidance of a doctor.)

(a−) 您好: 建议您及时到医院检查, 明确发热的病因, 积极退烧治疗. (Hello, it is recommended that you go to the hospital in time to check the cause of fever and actively treat fever.)

(a−) 你好, 经常拉肚子这种情况是脾肾虚寒导致的,建议服用四神丸治疗. (Hello, the symptom of diarrhea is often caused by spleen and kidney deficiency. It is recommended to treat it by taking Sishen Wan.)

**Table 2:** Advantages of pretraining method on Bi-LSTM.

Pretraining method	Train acc (%)	Train loss	Test acc (%)
Double-level	84.23	0.95	79.15
Character-level	80.75	1.47	77.23
Word-level	83.49	1.35	76.85

**Table 3:** Comparison of different similarity calculations.

Similarity	Train acc (%)	Train loss	Test acc (%)
Semantic similarity	84.23	0.95	79.15
Cosine similarity	81.85	0.647	71.83

**Table 4:** Performance on the insuranceQA dataset.

Model	DMN	Multiscale CNN	CapsNet	SCDE-Bi-LSTM
Train acc (%)	66.51	64.84	70.25	72.53

**Table 5:** Top-1 accuracy and loss of each model.

	Model	Train acc (%)	Train loss	Test acc (%)
1	BM25	44.80	∗∗∗	45.40
2	Multiscale CNN	66.53	1.95	64.67
3	Attentive pooling	85.33	1.7851	72.5256
4	DMN	75.24	0.92	74.38
5	ESIM + ELMo	80.37	1.15	77.15
6	Multiview	79.61	1.25	75.37
7	CapsNet	82.63	1.37	77.53
8	SCDE-Bi-LSTM	84.23	0.95	79.15

**Table 6:** Different performance measures on the test set.

	Model	*F*1 (%)	Recall (%)	Top-2 (%)	Top-3 (%)
1	BM25	49.02	53.27	49.62	52.73
2	Multiscale CNN	62.15	59.83	67.85	69.53
3	Attentive pooling	73.18	73.86	75.97	78.21
4	DMN	74.74	75.12	77.42	80.93
5	ESIM + ELMo	77.54	78.26	79.27	80.31
6	Multiview	76.09	76.83	78.14	79.83
7	CapsNet	78.17	78.82	80.61	83.53
8	SCDE-Bi-LSTM	79.83	80.52	82.13	84.73

**Table 7:** The number of steps taken by models.

Model	DMN	Convolutional LSTM	CapsNet	SCDE-Bi-LSTM
Steps	452	229	157	93

## Data Availability

The medical Q&A sentence data used to support the findings of this study have been deposited in the GitHub (https://github.com/Vitas-Xiong/Chinese-Medical-Question-Answering-System) repository.

## Supplementary Materials

Supplementary Materials In the supplementary material submitted along with the manuscript, the Graphical Abstract file is included. The file introduces center approach and main contributions in detail of this paper and describes the framework for question-and-answer matching in this paper.
