# Supplementary material for: IARNN-Based Semantic-Containing Double-Level Embedding Bi-LSTM for Question-and-Answer Matching
Source: Comput Intell Neurosci. 2019 Mar 3;2019:6074840. doi: 10.1155/2019/6074840 (PMC6421739; doi:10.1155/2019/6074840)
Supplement: Supplementary Materials — In the supplementary material submitted along with the manuscript, the Graphical Abstract file is included. The file introduces center approach and main contributions in detail of this paper and describes the framework for question-and-answer matching in this paper. [file 6074840.f1.pdf]

# IARNN Based Semantic-containing Double-level Bi-LSTM for Question Answer Selection

**Chang-zhu Xiong [1,*], Minglian Su [2]**

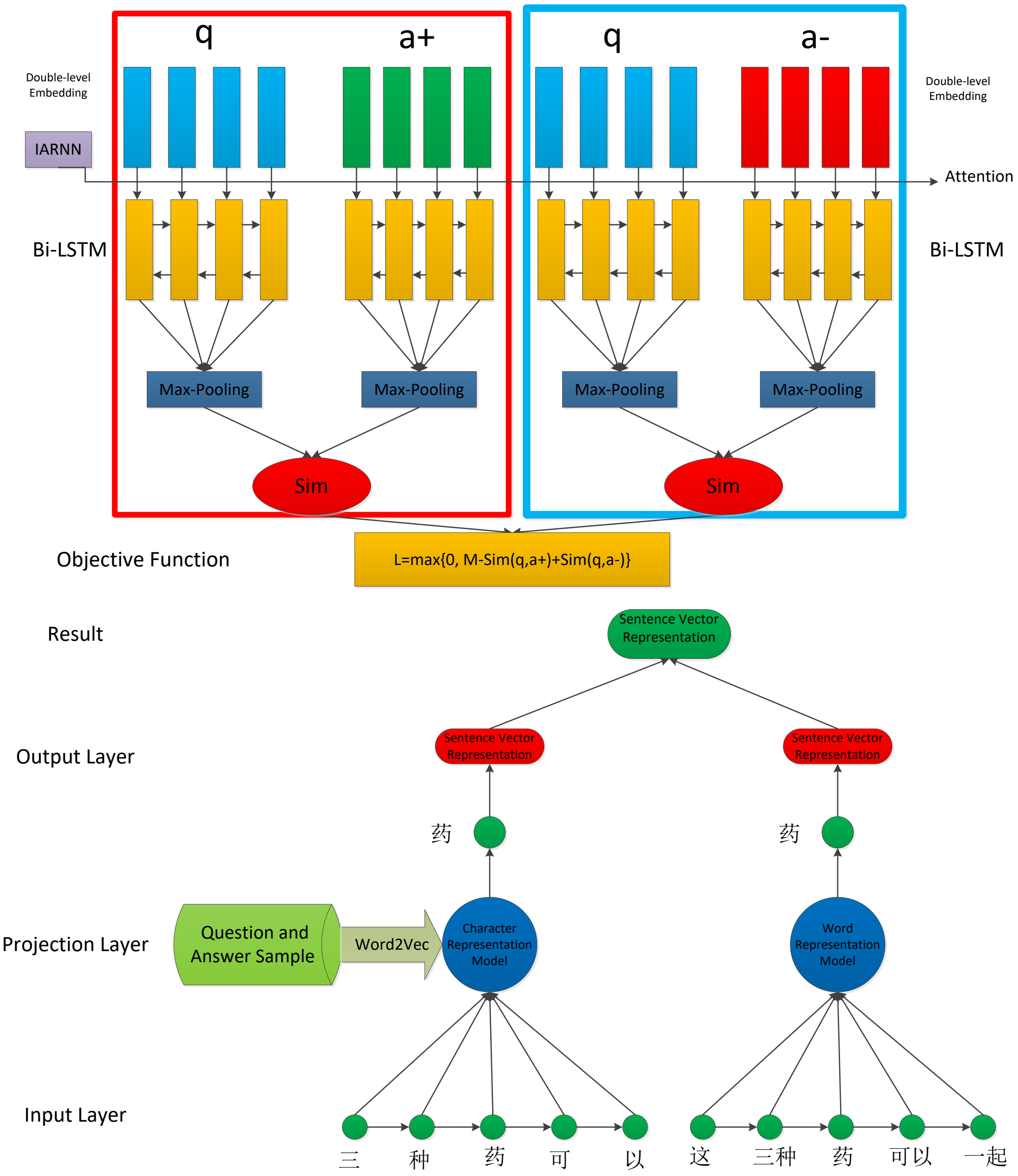


In this work, we solves Chinese word segmentation error by double-level embedding in sentence vector representation model, and solves the limitations of the traditional Q&A similarity calculation by using similarity calculation including semantic information. The proposed bidirectional LSTM framework based on the IARNN attention mechanism also successfully solves the problem of backward deviation of the traditional attention mechanism. The experimental results on real datasets exceed baseline accuracy by 14% and the accuracy of top-1 under Bi-LSTM can reach 79.15%, which has great potential for development and practical utility. Therefore, the research in this paper may have significant medical application impact.
